# Rheumatoid arthritis sera antibodies to citrullinated collagen type II bind to joint cartilage

**DOI:** 10.1186/s13075-022-02945-0

**Published:** 2022-11-22

**Authors:** Qixing Li, Yanpeng Li, Bibo Liang, Rui Xu, Bingze Xu, Erik Lönnblom, Hui Feng, Jing’an Bai, Roma Stawikowska, Changrong Ge, Aiping Lu, Gregg B. Fields, Lianbo Xiao, Rikard Holmdahl

**Affiliations:** 1 Center for Medical Immunopharmacology Research, Southern Medical University, Guangzhou, China; 2 Section for Medical Inflammation Research, Department of Medical Biochemistry and Biophysics, Karolinska Institute, Biomedicum, Quarter 9D, 171 65 Solna, Sweden; 3 Guanghua Integrative Medicine Hospital, Changning District, Shanghai, China; 4 Institute of Basic Research in Clinical Medicine, China Academy of Chinese Medical Sciences, Beijing, China; 5 Department of Chemistry & Biochemistry and I-HEALTH, Florida Atlantic University, Jupiter, FL USA; 6 The Second Affiliated Hospital of Xi’an Jiaotong University (Xibei Hospital), Xi’an, 710004 China

**Keywords:** Rheumatoid arthritis, Osteoarthritis, Anti-citrullinated type II collagen antibodies

## Abstract

**Objective:**

To investigate the occurrence and frequency of anti-citrullinated protein antibodies (ACPA) to cyclic citrullinated type II collagen (COL2) epitope with a capacity to bind joint cartilage.

**Methods:**

Luminex immunoassay was used to analyze serum antibody reactivity to 10 COL2-citrullinated peptides (ACC10) and corresponding arginine peptide controls in rheumatoid arthritis (RA), osteoarthritis (OA), and healthy individuals’ cohorts. Top ten “promiscuous” sera (cross-reactive with all ACC10) and top ten “private” sera (restrictedly reactive with one ACC10 peptide) from RA and OA cohorts were selected. Enzyme-linked immunosorbent assay (ELISA) was used to detect response to native COL2. Sera were analyzed with naive and arthritic joints from DBA/1J mice by immunohistochemistry, using monoclonal ACPAs and COL2 reactive antibodies with human Fc as comparison. Staining specificity was confirmed with C1 (a major antibody epitope on COL2) mutated mice and competitive blocking with epitope-specific antibodies.

**Results:**

All patient sera bound ACC10 compared with control peptides but very few (3/40) bound native triple-helical COL2. Most sera (27/40) specifically bound to arthritic cartilage, whereas only one private RA serum bound to healthy cartilage. Despite very low titers, private sera from both RA and OA showed an epitope-specific response, documented by lack of binding to cartilage from C1-mutated mice and blocking binding to wild-type cartilage with a competitive monoclonal antibody. As a comparison, monoclonal ACPAs visualized typical promiscuous, or private reactivity to joint cartilage and other tissues.

**Conclusion:**

ACPA from RA and OA sera, reactive with citrullinated non-triple-helical COL2 peptides, can bind specifically to arthritic cartilage.

**Supplementary Information:**

The online version contains supplementary material available at 10.1186/s13075-022-02945-0.

## Introduction

Rheumatoid arthritis (RA) develops through three stages: priming, onset, and chronicity [1]. In the priming stage, immune cells are activated, resulting in the production of disease-specific autoantibodies which can be detected in the blood several years before signs of clinical symptoms [2–5], including disease predictable autoantibodies like rheumatoid factors (RFs) [2] and anti-citrullinated protein antibodies (ACPAs) [3, 6, 7]. In the onset stage, joint pain and acute inflammation appears, which is followed by a chronic relapsing or progressive inflammatory disease, mainly in peripheral joints but also with systemic effect. The chronic stage has been extensively clinically characterized, concluding that the disease is associated with a destructive joint inflammation with increased serum autoantibody levels, but the origin, specificity, and function of these reactivities remain largely unknown [8, 9].

It has been reported that ACPAs can react with many different proteins like vimentin, α-enolase, fibrinogen, filaggrin, and type II collagen (COL2) [10–13]. ACPAs are highly specific for a citrulline side chain in the center of an epitope [14]. In RA, autoantibodies against citrullinated epitopes on major articular cartilage protein COL2 occur in serum and synovial fluid [15]. Antibodies to citrullinated and native triple-helical forms of COL2 binds to joint cartilage [16, 17], but it is not known whether the more common ACPAs cross-reacting with citrullinated alpha chain epitopes on COL2 [18] can bind to cartilage. To understand the specific role of different antibodies, we have analyzed several monoclonal ACPAs from both mice and RA patients and suggested a classification of the different ACPAs based on their structural interactions with their target citrullinated epitopes [17]. On one end are *promiscuous ACPAs*, which refer to ACPA that can specifically bind to the citrullinated side chain and only negatively interact with adjacent side chains. On the other end are *private ACPAs*, which recognize specific citrullinated epitopes but also adjacent amino acid side chains. Private ACPA divide into *private specific* and *private cross-reactive*. The former can specifically recognize citrulline located in the central CDR3 forming pocket, whereas the latter interact with the citrulline sidechain outside the central region.

B cells producing RF and ACPA are most likely primed in mucosal sites and not in joints [19–21]. Interaction with T cells will lead to IgG production. With time, new B cell clones with more restricted reactivities may be expanded, some of them with a higher epitope specificity, i.e., more private reactivity [17]. Although the origin and function of this diversification of ACPA reactivities are unknown, it is of interest that some cross-react to joint cartilage, and with COL2 [16, 22, 23]. This is of interest since only antibodies binding cartilage in vivo is known to induce arthritis in experimental systems [24–26]. Monoclonal antibodies binding citrullinated triple-helical COL2 can induce and enhance arthritis [22]. In established RA, antibodies to triple-helical COL2 rarely occur but seem to be more frequent in an early stage of the disease and synovial fluid [27, 28]. However, reactivities to cyclic citrullinated COL2 alpha chain peptide epitopes are more common [12, 18], but their degree of specificity and capacity to bind cartilage are unknown. Osteoarthritis (OA) is another common joint disease, which differs from RA, but is very heterogenous including some variants being more inflammatory and others more degenerative [29]. Autoantibodies, including ACPAs and anti-COL2 antibodies, may also occur in serum and synovial fluid from some OA patients [30, 31]. Although ACPAs are one of the hallmarks in serological test in RA diagnosis, the border is not clear and antibodies to citrullinated COL2 may overlap between the two entities.

We have developed a bead-based multiplex assay kit in which all major citrullinated COL2 epitopes are included as cyclic peptides in a previous study [18]. A cohort including 415 established-RA and 304 established-OA patients were used to detect this series of cyclic peptides, using 203 healthy controls for comparison. We selected the 10 most commonly targeted peptides (denoted the ACC10 test). We could show that 83.5% of the RA and 14.8% of the OA patients had antibodies to ACC10, consisting of these selected cyclic citrullinated COL2 peptides.

We have now analyzed the capacity of the sera to bind to naïve and arthritic cartilage. As all the investigated B cell epitopes to COL2 are conserved between mouse and human [32], we used well-controlled mouse joint tissues for the analysis. With a mouse strain mutated at a major COL2 epitope (C1), and with competing monoclonal antibodies, we could also show that antibodies from both RA and OA sera bind specific COL2 epitopes in cartilage.

## Methods

### Study subjects

In this study, RA and OA patient’s sera came from Shanghai Guanghua Hospital and healthy individuals’ sera were from the Chinese Academy of Medical Science (described in [18]). The diagnosis of RA follows the 1987 American College of Rheumatology criteria [33]. The diagnosis of OA is followed by the R. Altman proposed classification criteria in 1987 [34].

### Bead-based multiplex immunoassay

A bead-based multiplex Luminex-based immunoassay has been used to analyze sera antibody binding to cyclic and triple-helical COL2 peptides (THP) [18, 35]. Sera were diluted at a ratio of 1:100. The median fluorescence intensity (MFI) was used to quantify the interaction of serum antibodies with the given peptides. The sequences of cyclic peptides and THPs are shown in Supplementary Tables S1 and S4, respectively. All COL2 peptides included in the study were highly conserved between mouse and human. Three of the peptides contained an amino acid with a difference but these are not likely influencing binding (Table S1).

Based on the results of the bead-based multiplex assay and the suggested classification of ACPA mentioned above, we defined sera that show promiscuous antibody reactivity overall as promiscuous sera, denoted pro-RA, and pro-OA to facilitate comparison. Another extreme type of sera reacting with one cyclic citrullinated COL2 peptide was defined as private sera, denoted pri-RA, and pri-OA. Ten patient sera in each group were selected for closer analysis of their specificity, and 5 sera selected from the healthy control group, were used as negative controls, denoted as HC (Table S2).

### Enzyme-linked immunosorbent assay (ELISA)

To detect triple-helical COL2 antibodies, 5 mg/ml bovine COL2 was used for coating on ELISA plates. PBS with 0.05% tween20 was used to wash the plates and 3% milk powder as blocking reagent. As primary antibodies, mAbs were diluted to 5 mg/ml, and selected sera diluted 40X. Goat-anti-human IgG HRP conjugated antibody (Southern Biotech, 3048-05) was used as the secondary antibody and TMB for developing the color reaction. The OD value was determined at l = 450 nm.

### Anti-CCP test

Anti-CCP2 antibody levels in RA patients’ serum were measured using the standard anti-CCP2 antibody kit (EuroDiagnostica, RA-96PLUS kit, Sweden). Absorbance value (optical density) ratio were calculated to measure the anti-CCP2 reacitvity for the positive and negative controls and for each sample.

$$Absorbance\ Ratio=\frac{\textrm{Sample}\ \textrm{or}\ \textrm{Control}\ \textrm{Absorbance}\ \textrm{Value}}{\textrm{mean}\ \textrm{Reference}\ \textrm{Control}\ \textrm{Absorbance}\ \textrm{Value}}$$

Results from the patient populations used in the Svar Life Science clinical trial suggest the following cut-off:

**Table:** 

Absorbance ratio	Results interpretation
< 0.95	Negative
≥ 0.95 to ≤ 1.0	Borderline—recommend repeat testing
> 1.0	Positive

### Animal models

DBA/1J mice, 8–10 weeks old, were used for the collagen-induced arthritis (CIA) model, which were scored using an established protocol based on 1 score per inflamed joint (each paw ranges from 0 to 15) [28]. To detect mAbs in vitro reactivity, we used murine joints from neonatal C57BL/6 mice (3 days old), following a previously described protocol [25]. As a source of joints with established arthritis, we used mice with CIA (obtained at D90 after first immunization). Skin from naïve mice or inflamed skin (obtained at D5 after immunization) from psoriatic mice (from mannan-induced psoriasis (MIP) model [36]) and naïve Wistar rat esophagus were collected to obtain cryosections for immunohistochemistry (IHC) staining. At the same time, mice with a targeted mutation within the C1 epitope of COL2 (replacing Arg360 with Gln) was used with the CIA model to obtain arthritic joint tissue at D90 after first immunization (Rui et al., unpublished data). All paws were decalcified, dehydrated, and embedded to make cryosections at 8 mm thickness.

### Histology

For IHC staining, cryosections were used to ascertain protection of antigen epitopes. Primary antibodies (5 mg/ml mAbs and sera diluted to 40X) were incubated for 40 min at RT. All the sections were incubated with anti-human IgG HRP conjugated secondary antibody (Southern biotech, 6140-05) for 30 min at RT and stained with DAB solution (VECTOR, SK-4100) for 3 min in dark environment. Then, slides were counterstained with hematoxylin for 90 s. Inverted microscope (Leica DM500 (LAS V4.9)) was used to capture the sections. For analysis, Image-pro Plus 6.0 was used to measure MOD (mean of integrated optical density) to evaluate the IHC staining strength with × 400 magnification.

### Statistics

One-way ANOVA and Mann-Whitney *U* test were applied to analyze the difference in serum antibody responses to triple-helical COL2 peptides and other antigen peptides. Paired *T* test was applied to analyze the difference in serum antibody response to ACC10 and corresponding arginine peptides.

## Results

### ACPAs and joint reactive antibodies show different reactivity to joint tissue

**Fig. 1:**
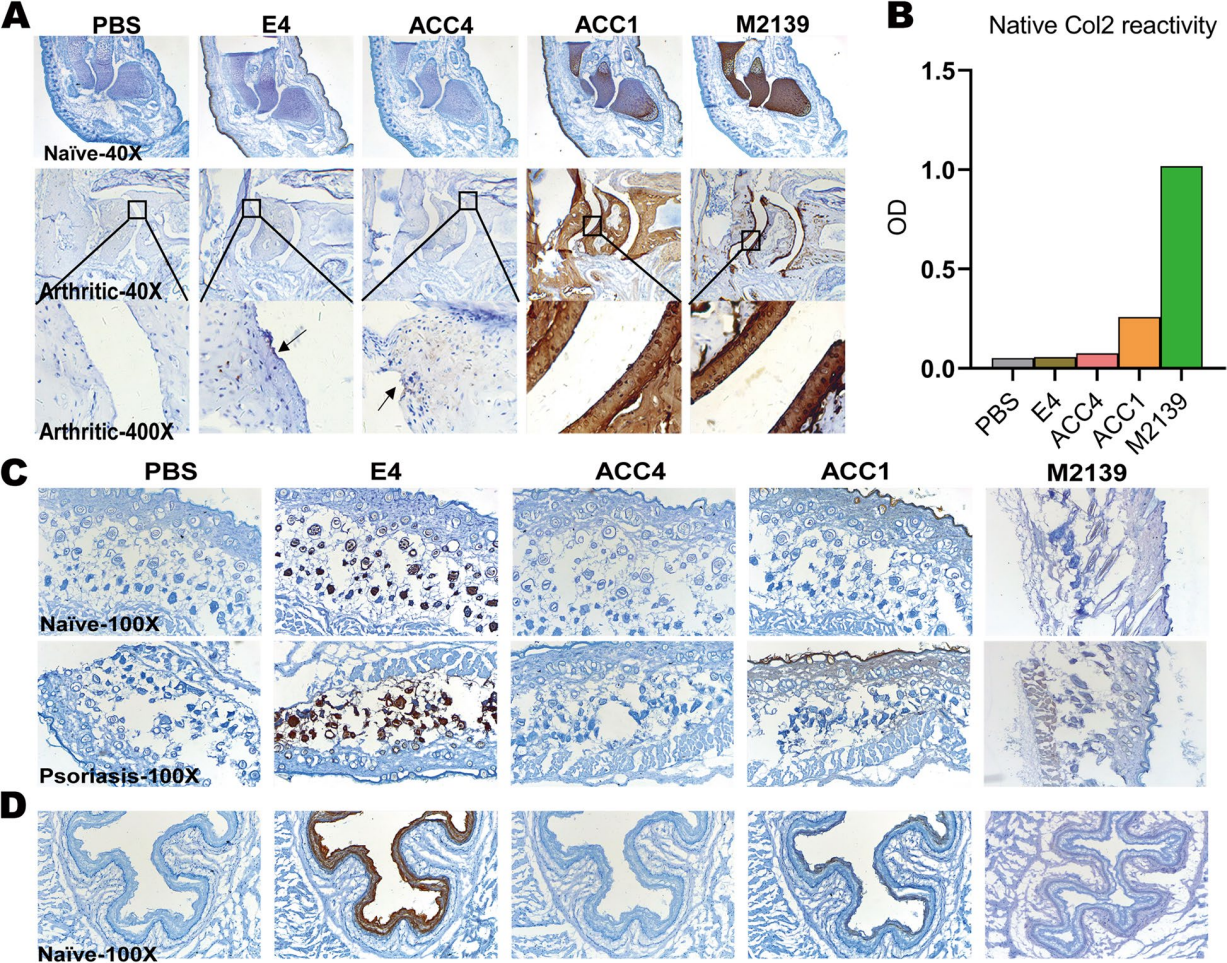
The reactivity of monoclonal ACPAs and COL2 reactive antibodies in vitro. **A** Antibody binding to joint tissue from naïve neonatal joints and arthritic joints obtained at D90 after immunization (historic maximum arthritis score = 13). **B** Antibody response to native triple-helical COL2 by ELISA detection. **C** Antibody in vitro reactivity to skin tissue from naïve mice and inflamed skin of mice with psoriasis at D5 after immunization. **D** Antibody in vitro reactivity to naïve Wistar rat esophagus. PBS is a negative control. × 40, × 100, and × 400 are the magnification of captured pictures

We characterized naïve neonatal joint and arthritic joint tissues using a series of well-defined monoclonal antibodies, listed in Table 1. We found that only ACC1 (*private cross-reactive ACPA*) and M2139 (*joint reactive antibody*) gave a strong specific staining of cartilage from both naïve and arthritic joints (Fig. 1A) and could detect native COL2 (Fig. 1B). Both *promiscuous* E4 and *private specific* ACC4 showed no reactivity to native COL2 or cartilage in naïve joints but had a weak reactivity to arthritic joint cartilage. The *private specific* ACC4 could also bind to arthritic joint synovial tissue (Supplementary Fig. S1).

**Table 1:** The specificity information of different monoclonal antibodies in this study

Antibodies	Origin	Classification	Isotype	Specificity
E4	Chimeric antibody isolated from RA patients	Promiscuous	hIgG1	Reactive with several citrullinated peptides, including citrullinated F4 derived from COL2 [14]
ACC4	Triple-helical citrullinated C1 epitope immunized mice	Private specific	hIgG1	Specific for the alpha chain of citrullinated C1 epitope on COL2 [22, 23]
ACC1	Triple-helical citrullinated C1 epitope immunized mice	Private cross-reactive	hIgG1	Reactive with citrullinated C1 epitope on COL2 but also cross-reactive with several epitopes on triple-helical COL2 [22, 23]
M2139	COL2 immunized mice	Joint reactive	hIgG1	Specific for the J1 epitope on triple-helical COL2 [37]

### The cross-reactive ACPAs bind to skin endothelial cells and esophagus epithelial cells

In naive joint IHC, both *promiscuous* E4 and *private cross-reactive* ACC1 bound to the skin covering the joints (Fig. 1A). We, therefore, tested the in vitro reactivity and binding specificity of this series of antibodies to healthy skin, inflamed psoriatic skin (Fig. 1C), and esophagus from healthy Wistar rats (Fig. 1D). We found that E4 bound to keratinocytes in the dermal layer of healthy and inflammatory skin and rat esophageal epithelial cells. ACC1 preferentially bound to the cartilage but also the bone in the joints (Fig. 1A), the epidermis or corneum of healthy and inflammatory skin, and rat esophageal mucosal epithelia. The *private specific* ACC4 and *joint reactive* M2139 showed no reactivity to the bone, skin, or esophagus.

### Serum autoantibodies show reactivity and relative specificity to cyclic citrullinated COL2 peptides in vitro

**Fig. 2:**
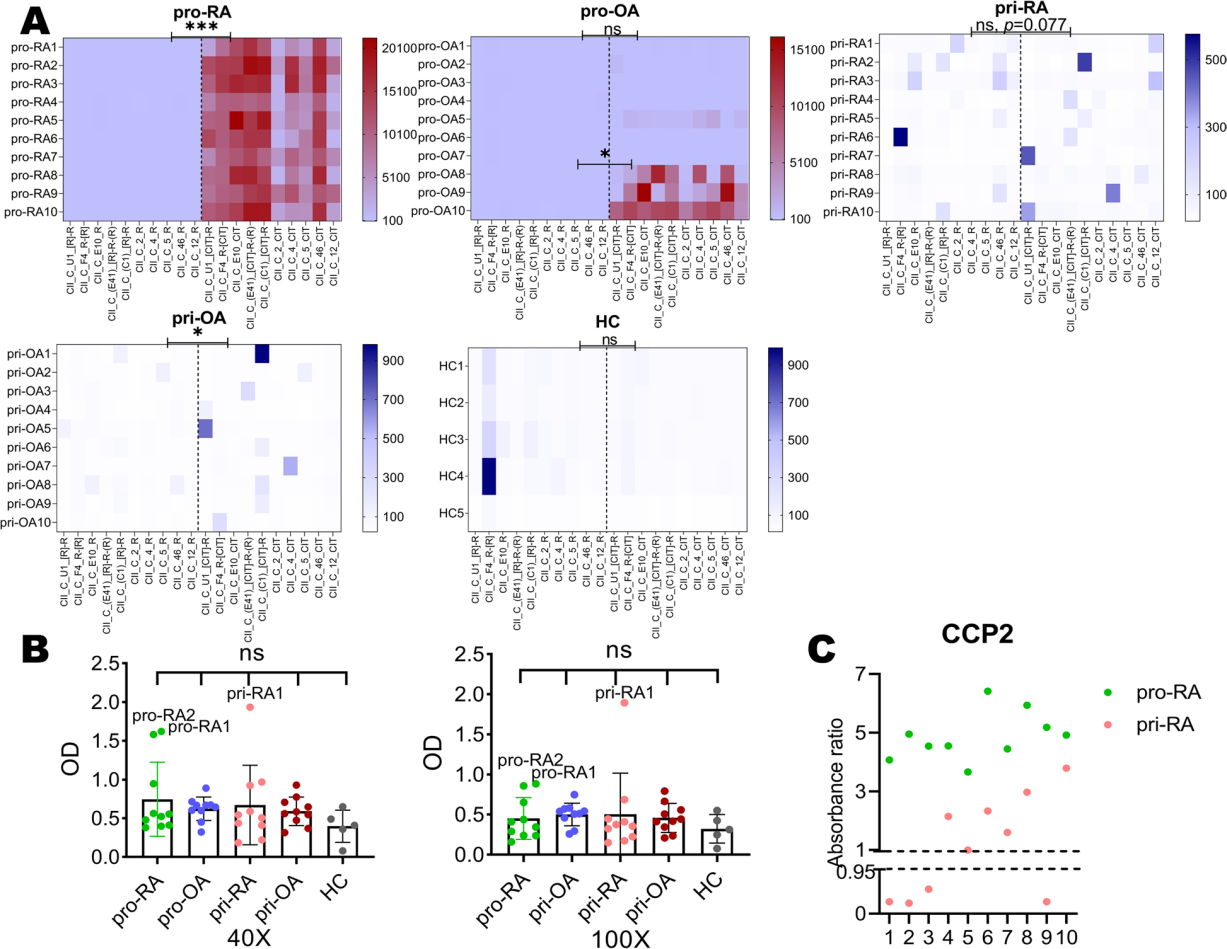
The sera reactivity to cyclic COL2 peptides and native triple-helical COL2. **A** Promiscuous sera showed higher reactivity to ACC10 while the private showed higher specificity but relatively low reactivity. Dash line separates arginine peptides (left) and ACC10 (right). ***P < 0.001, *P < 0.05; ns, no significance. **B** Sera response to native triple-helical COL2 in × 40 and × 100 diluted ratio. **C** Anti-CCP test for RA patients was detected using commercial EuroDiagnostica, RA-96PLUS kit. Absorbance ratio < 0.95 is defined as negative and > 1.0 as positive. pro-, promiscuous; pri-, private; HC, healthy individuals sera

The sera response of both private groups to citrullinated COL2 peptide was much lower than that of promiscuous (Fig. 2A). It is likely that RA sera classified as promiscuous show strong reactivity because of the pronounced cross-reactivity of the antibodies. Pro-RA sera showed much stronger reactivity to ACC10 than pri-RA sera (Fig. 2A). Only a few of OA sera had reactivity to citrullinated peptides and the reactivities were generally lower compared with RA sera (Fig. 2A). Antibody response to corresponding arginine peptides of ACC10 were lower than response to ACC10, but only pro-RA and pri-OA showed significant binding in comparison. Pro-OA showed difference only when compared with strong ACC10 positive samples, including pro-OA8, pro-OA9, and pro-OA10 (Fig. 2A). A few RA patient sera (3/40) showed reactivity to native COL2 in ELISA detection (Fig. 2B). It is worth mentioning that pri-RA1 could still detect native COL2 even in a low diluted ratio such as 1:100 (Fig. 2B). All pro-RA sera and a half (5/10) of pri-RA sera were CCP2 positive (Fig. 2C). Of the OA patients and healthy individuals’ cohort used in our previous study [18], 96.4% OA and 98% healthy individual samples were CCP2 negative.

### Promiscuous sera show reactivity to many citrullinated cyclic and triple-helical peptides

**Fig. 3:**
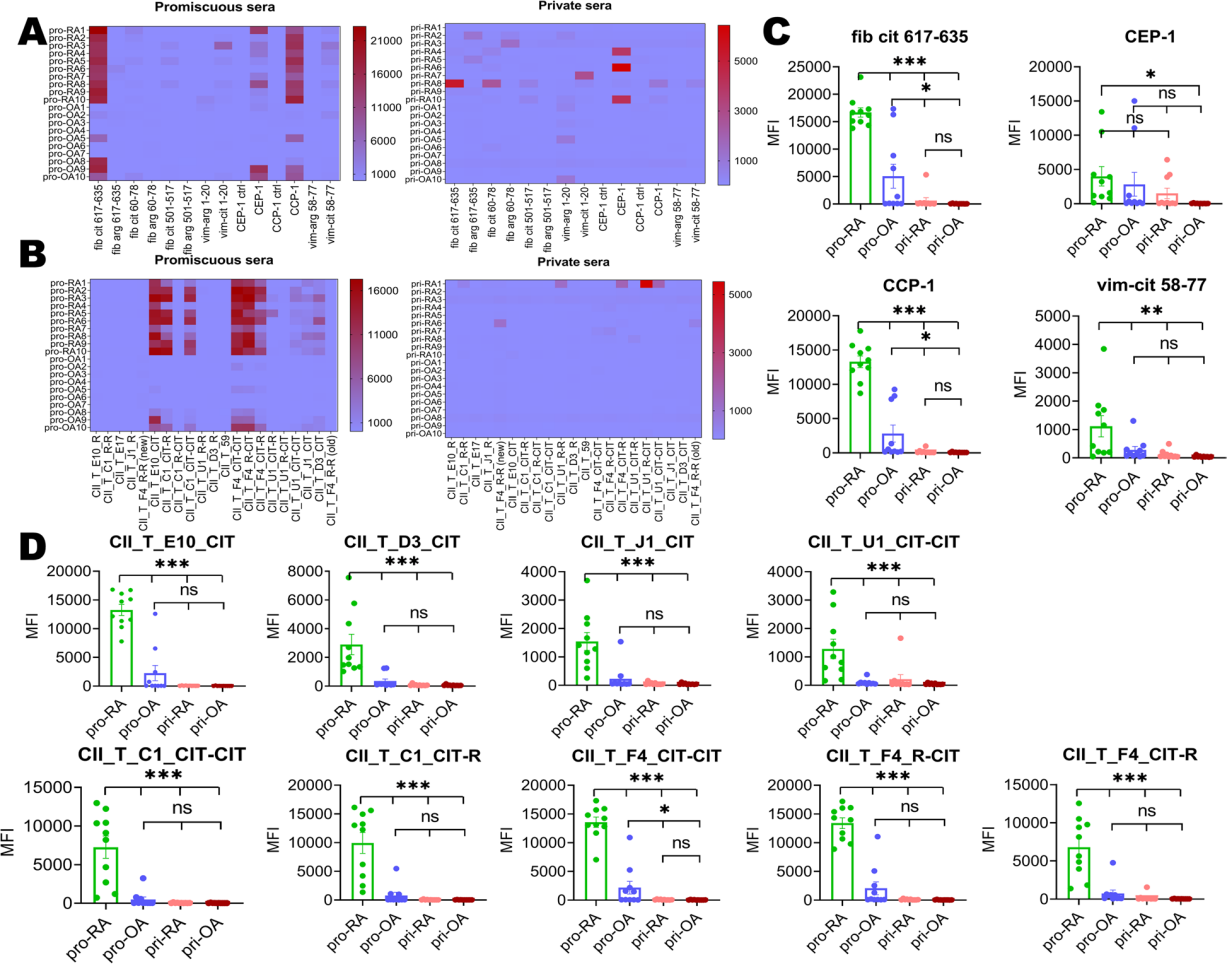
Sera presented different reactivity to other antigen peptides (**A**) and triple-helical COL2 peptides (**B**). Promiscuous sera showed a higher reactivity to *fib cit 617-635*, *CEP-1*, *CCP-1*, and *vim cit 58-77* compared with the private (**C**). As for triple-helical COL2 peptides (**D**), promiscuous sera also show cross-reactivity to citrullinated E10, D3, J1, U1, C1, and F4 petides. Private sera almost had no reactivity to triple-helical COL2 peptides except for pri-RA1. ***P < 0.001, **P < 0.01, *P < 0.05; ns, no significance. pro-RA, promiscuous RA sera; pro-OA, promiscuous OA sera; pri-RA, private RA sera; pri-OA, private OA sera

In terms of the reaction of serum antibodies to other antigen peptides (apart from COL2 peptides), promiscuous sera were generally more reactive than private sera (Fig. 3A, B). Pro-RA sera showed binding to citrullinated fibrinogen, citrullinated a-enolase, citrullinated filaggrin, and citrullinated vimentin peptides (Fig. 3C, peptide information in Table S3). The pro-RA sera showed binding to several citrullinated THPs with identified epitopes (Fig. 3B, D). As for private sera, only pri-RA1 could bind to THPs with a high response, where the corresponding epitopes were arginine U1, citrullinated U1, and citrullinated F4.

### Most patient sera bind to arthritic joint cartilage, but few can bind to naïve joints

**Fig. 4:**
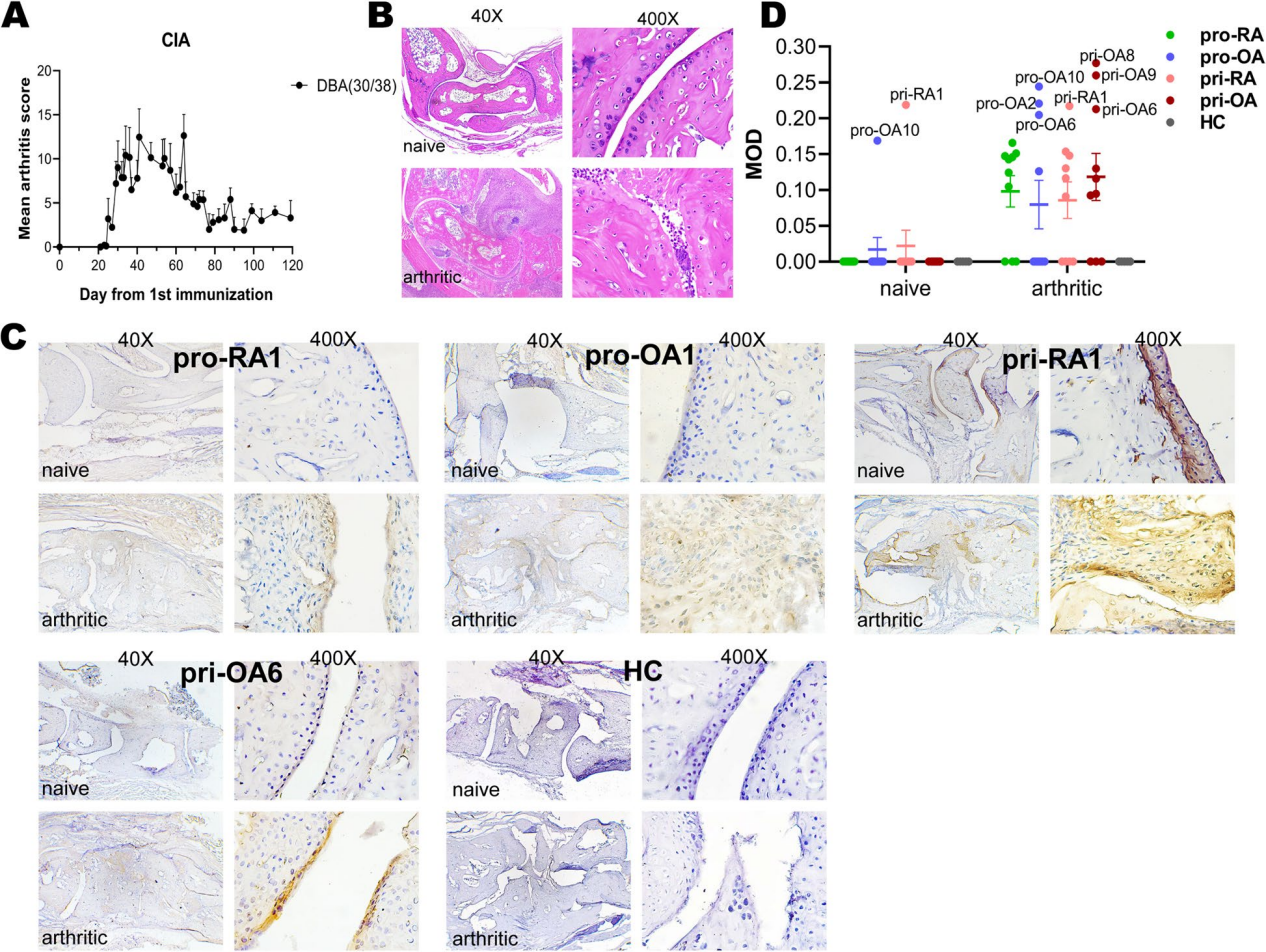
IHC staining results of promiscuous and private groups. **A** Mean arthritis score (0–60) of the CIA experiment with DBA1/J. **B** Hematoxylin-eosin staining of naïve mouse hind paw and arthritic DBA1/J hind paw (arthritis score > 12). **C** Representative IHC staining results from promiscuous, private and HC sera. **D** MOD is a parameter to measure the in vitro reactivity of sera to naïve and arthritic joint tissue. 40X, 400X: the magnification of pictures. MOD: mean of integrated optical density. pro-RA: promiscuous RA sera; pro-OA: promiscuous OA sera; pri-RA: private RA sera; pri-OA: private OA sera; HC: healthy individuals sera

We obtained normal healthy joints from naïve DBA1/J male mice and established arthritis joints from mice at day 90 after first COL2 immunization (Fig. 4A) with a historic peak arthritis score > 12 for each hind paw (Fig. 4B). The IHC results showed that 7/10 pro-RA, 4/10 pro-OA, 6/10 pri-RA, and 7/10 pri-OA could bind to arthritic joint cartilage or synovial pannus tissue whereas only pro-OA10 and pri-RA1 could bind to naïve joint. Private sera showed a stronger and more specific reactivity to joint cartilage as well as to synovial tissue than the promiscuous sera (Fig. 4C, D). Sera reactivity to joints were quantified and outstanding sera indicated (Fig. 4D). The in vitro reactivity of pri-RA1 to naïve joint and arthritic joint is in accordance with its response to native COL2, ACC10, and THPs as described above. The pro-OA10 showed no reactivity to native COL2 in ELISA detection but some reactivity to both naïve and arthritic joint tissue.

### Private sera contain antibodies detecting cartilage through specific epitopes

**Fig. 5:**
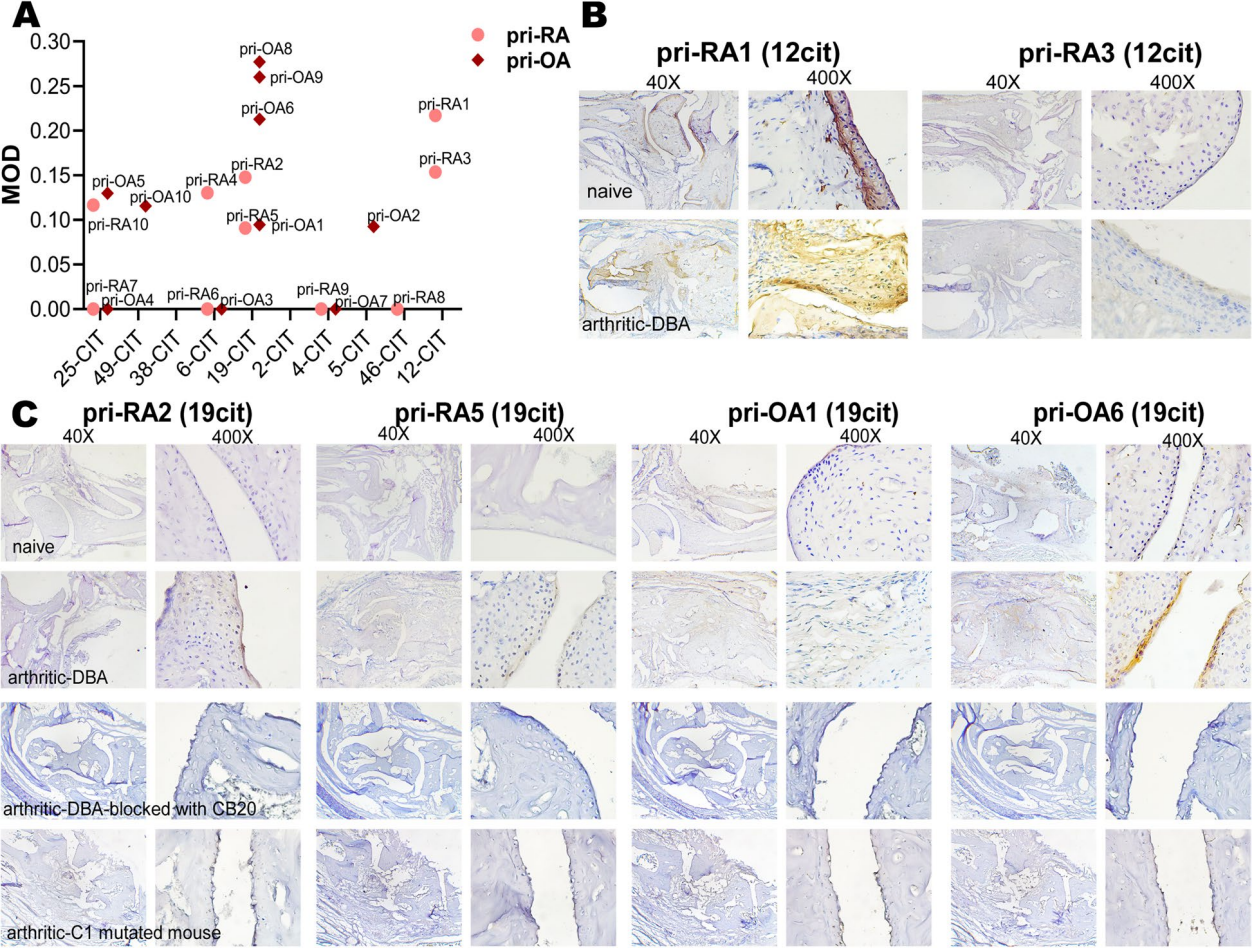
Private sera showed specific binding to cartilage in vitro. **A** Private sera reactivity to arthritic DBA1/J joint obtained at D90 after 1st immunization (arthritis score > 12) tissue. The *x*-axis shows the citrulline sites of sera responded peptides. **B** IHC results of sera that had private response to 12-cit cyclic COL2 peptide. **C** IHC results of sera that had private response to 19-cit cyclic COL2 peptide (specific to C1 epitope). The sera did not bind to arthritic joint tissue listed: (a) obtained at D90 after immunization from C1 mutated mouse (historic max arthritis score = 14) and (b) arthritic DBA joint tissues as described previously but blocked with 5 mg/ml CB20 before sera incubation

ACC10 and their corresponding citrulline sites to which private sera bind are listed in Supplementary Table S5. The private sera are mainly reactive with 25cit and 19cit peptides, corresponding to citrullinated U1 and C1 epitopes on COL2, respectively. The reactivity of private serum antibodies to arthritic joint tissue were quantified with MOD values (Fig. 5A). We found that sera with more specific reactivity to 19cit and 12cit peptides had outstanding reactivity to arthritic joint tissues. In the private group, only pri-RA1(12cit) could react with native COL2, and this reactivity was also observed with naïve joint cartilage. Another private sera pri-RA3(12cit) responded to the same cyclic citrullinated peptide as pri-RA1(12cit), but the reactivity to joint tissue was much weaker (Fig. 5B). One possible explanation is that pri-RA1(12cit) had a strong response to several THPs with U1 and F4 epitopes on COL2, but pri-RA3(12cit) showed no reactivity to the THPs. As for 19cit specific private sera, although they were less reactive to cyclic citrullinated peptides than promiscuous sera (Fig. 2A), they presented stronger and more specific binding in vitro. 19cit is the citrulline site where the C1 epitope is located on the COL2. We confirmed that sera with reactivity to C1 citrullinated peptides contained antibodies binding to the C1 epitope by using CB20, a C1 epitope-specific monoclonal antibody, to block the C1 epitope on the joint tissue (Fig. 5C). We also compared the IHC results of these 19cit specific private sera with arthritic joint tissues of mice with a mutation of the C1 epitope, where the essential amino acid R360 was replaced. Combined with in vitro results, we conclude that 19cit peptide reactive private sera specifically bind to the C1 epitope of arthritic joint tissues.

## Discussion

A key question to solve in understanding why RA develops is to determine if emerging ACPAs can switch to be reactive to peripheral joints. We found that sera from most RA, and even some OA patients, contained antibodies that could bind to arthritic joint cartilage. Even sera containing antibodies which only weakly, but more restrictedly, detected citrullinated COL2, could bind joint cartilage. Thus, we conclude that even low titers of private ACPA detecting citrullinated COL2 could bind to joint cartilage.

Previous bead-based immunoassay results [18] showed that 81% of RA sera (336/415) and 1.97% of OA sera (6/304) could be classified as promiscuous, whereas 2.41% of RA sera (10/415) and 4.6% of OA sera (14/304) were classified as private. In this study, we also included ACC10 positive OA sera to investigate for comparison. Antibodies reactivity to citrullinated COL2 in OA development has not been well clarified [31]. Our study verified that antibodies in ACPA positive OA sera could bind to arthritic joint cartilage.

Compared with CCP2 test, using ACC10 to detect the same OA cohort raised the positive rate (from 3.6 to 14.8%) of OA sera to citrullinated COL2 peptides. Interestingly, private OA sera (4.6%, 14/304) presented a higher prevalence than promiscuous OA (1.97%, 6/304). We also found that some CCP2 negative RA sera showed specific and strong binding to joint cartilage, indicating that antibodies of these CCP2 negative patients may also attacking the cartilage in RA. Thus, this could enlighten results from previous studies, in which a subset of ACPA-positive patients were CCP2-negative [38, 39]. A novel finding of our study is that ACC10 multiplex immunoassay is more sensitive and specific than the CCP2 test to detect antibodies reactive with citrullinated COL2, thereby potentially binding to joint cartilage.

It has been known for a long time that autoantibodies, both RF and ACPA, occur years before the onset of RA [2], but their function or relation to the later joint inflammatory attack and their role for RA development have still not been clarified. Interestingly, the tipping point for arthritis development may not be when ACPA first appear [40] but rather at a stage with quality shift, such as glycosylation [41, 42], or specificity changes occurring predominantly in individuals with certain MHC class II alleles [43]. It has widely been assumed that most autoantibodies are pathogenic since they are associated with disease development and because depletion of B cells is therapeutic [44]. Likewise, ACPAs are believed to be pathogenic based mainly on in vitro activation of various inflammatory cells. However, the only conclusive evidence is the induction of arthritis after transferring antibodies into mice, which also includes bone erosions and pain. These effects are mediated only by antibodies with reactivity to joints, binding to the cartilage surface [22, 24, 26, 45–47]. Thus, it is of importance to understand how a cross-reactive specificity pattern of “promiscuous” ACPA could develop into a more “private” specificity with the potential to bind to joint cartilage.

Interestingly, all three classes (promiscuous, private specific, and private cross-reactive) of ACPAs, represented by monoclonal antibodies, showed restricted binding to tissues, and both the promiscuous E4 and the private cross-reactive ACC1 antibodies behaved as classical ACPAs, in that they bound to rat esophageal epithelia.

ACC10 used to screen patient cohorts in a previous study [18] were used to screen promiscuous and private sera and validate their promiscuous or private reactivity to different conformations of COL2 and other antigens in this study. It was remarkable that the tissue specificity of the frequently occurring promiscuous ACPA was inferior to sera with private ACPA with lower titers and less reactive antibodies. The functional relevance of the used representative monoclonal antibodies have been described in previous studies [14, 22, 23, 48]. We suggest that antibodies in private RA sera, like the 19cit specific private serum antibodies, may have a similar arthritogenic effect as ACC4.

Antibodies to several epitopes on COL2 can be detected in RA [28, 32, 35], although at much lower frequency than ACPA or RF, and monoclonal antibodies to these COL2 epitopes are arthritogenic. Therefore, antibodies occurring in sera might represent a fraction of antibodies that could interact with cartilage and thereby enhance or regulate the development of arthritis.

Antibodies reactive with cyclic citrullinated COL2 peptides detected arthritic cartilage rather than naïve healthy cartilage, probably due to the citrullination and destabilization of COL2 in the arthritic joints. We previously found that antibodies to citrullinated cyclic epitopes can occasionally cross-react with native triple-helical epitopes [23], although it is likely that the present target epitopes are flexible, citrullinated and partially denatured from the triple-helical structure, in order to enhance interaction with the antibodies.

Our data indicates that RA and OA serum antibodies reactive with citrullinated COL2 can have the capacity to bind to joint cartilage, although it remains to be investigated whether this binding have any functional consequences regarding arthritis, bone erosions, or pain. To address the functionality of the antibodies, monoclonal antibodies are needed. We have previously shown that monoclonal antibodies binding cartilage elicit mechanical hypersensitivity and arthritis [47] whereas monoclonal antibodies against the citrullinated triple-helical C1 epitope of COL2 also bind cartilage and induce arthritis [23]. We previously found that serum ACPA directed to the citrullinated triple-helical F4 epitope (citrullinated at either Arg^927^ or Arg^933^) bound to RA cartilage [16]. The present study expands on this work by demonstrating that antibodies to cyclic citrullinated COL2 epitopes, a common reactivity in RA sera, can target cartilage. The present findings also strengthen the need for determining the fine specificity of the antibody response in RA, as this potentially could be important for classification of RA, allowing better diagnosis and therapy decisions.

## Supplementary Information

**Additional file 1: Supplementary Table S1.** Cyclic human COL2 peptide sequences (ACC10 and their corresponding arginine peptides). Amino acids differing from mouse are indicated in italic. **Supplementary Table S2.** Detailed information of antibody response to ACC10 in different sera subset. **Supplementary Table S3.** Information of other antigen peptides. **Supplementary Table S4.** Triple-helical COL2 peptide sequences. **Supplementary Table S5.** Peptides to which private sera have specific responses. **Fig. S1.** Some characteristics from patients with promiscuous or private ACPA. **Supplementary Figure S2.** Monoclonal ACPA and COL2 reactive antibody in vitro binding to newly developed arthritic joint tissue (arthritic score=13).

## Data Availability

The datasets used and/or analyzed during the current study are available from the corresponding author on reasonable request.
