# Supplementary material for: Rheumatoid arthritis sera antibodies to citrullinated collagen type II bind to joint cartilage
Source: Arthritis Res Ther. 2022 Nov 22;24:257. doi: 10.1186/s13075-022-02945-0 (PMC9682822; doi:10.1186/s13075-022-02945-0)
Supplement: Supplementary file 1 — Additional file 1: Supplementary Table S1. Cyclic human COL2 peptide sequences (ACC10 and their corresponding arginine peptides). Amino acids differing from mouse are indicated in italic. Supplementary Table S2. Detailed information of antibody response to ACC10 in different sera subset. Supplementary Table S3. Information of other antigen peptides. Supplementary Table S4. Triple-helical COL2 peptide sequences. Supplementary Table S5. Peptides to which private sera have specific responses. Fig. S1. Some characteristics from patients with promiscuous or private ACPA. Supplementary Figure S2. Monoclonal ACPA and COL2 reactive antibody in vitro binding to newly developed arthritic joint tissue (arthritic score=13). [file 13075_2022_2945_MOESM1_ESM.docx]

**Supplementary Tables**

Supplementary Table S1. Cyclic human COL2 peptide sequences (ACC10 and their corresponding arginine peptides). Amino acids differing from mouse are indicated in italic.

| Name | Epitope | Sequence |
| --- | --- | --- |
| CII_C_U1_[CIT]-R | U1_1 | Biotin-Ahx-CAPGLVGPcGERGFPGCA |
| CII_C_F4_R-[CIT] | F4_2 | Biotin-Ahx-CERGLKGHcGFTGLQGCA |
| CII_C_E10_CIT | E10 | Biotin-Ahx-CPQGLAGQcGIVGLPGCA |
| CII_C_(E41)_[CIT]-R-(R) | E41_1 | Biotin-Ahx-CSPGPMGPcGLPGERGCA |
| CII_C_(C1)_[CIT]-R | C1_1 | Biotin-Ahx-CEPGLPGAcGLTGRPGCA |
| CII _C_2_CIT |  | Biotin-Ahx-CVSGPMGPcGPPGP*P*GCA |
| CII _C_4_CIT |  | Biotin-Ahx-C*P*PGPQGAcGFPGTPGCA |
| CII _C_5_CIT |  | Biotin-Ahx-CLPGVKGHcGYPGLDGCA |
| CII _C_46_CIT |  | Biotin-Ahx-CPSGPAGAcGI*Q*GPQGCA |
| CII _C_12_CIT |  | Biotin-Ahx-CAPGFPGPcGPPGPQGCA |
| CII_C_U1_[R]-R | U1_1 | Biotin-Ahx-CAPGLVGPRGERGFPGCA |
| CII_C_F4_R-[R] | F4_2 | Biotin-Ahx-CERGLKGHRGFTGLQGCA |
| CII_C_E10_R | E10 | Biotin-Ahx-CPQGLAGQRGIVGLPGCA |
| CII_C_(E41)_[R]-R-(R) | E41_1 | Biotin-Ahx-CSPGPMGPRGLPGERGCA |
| CII_C_(C1)_[R]-R | C1_1 | Biotin-Ahx-CEPGLPGARGLTGRPGCA |
| CII _C_2_R |  | Biotin-Ahx-CVSGPMGPRGPPGP*P*GCA |
| CII _C_4_R |  | Biotin-Ahx-C*P*PGPQGARGFPGTPGCA |
| CII _C_5_R |  | Biotin-Ahx-CLPGVKGHRGYPGLDGCA |
| CII _C_46_R |  | Biotin-Ahx-CPSGPAGARGI*Q*GPQGCA |
| CII _C_12_R |  | Biotin-Ahx-CAPGFPGPRGPPGPQGCA |

Supplementary Table S2. Detailed information of antibody response to ACC10 in different sera subset

| Group | pro-RA | pro-OA | pri-RA | pri-OA | HC |
| --- | --- | --- | --- | --- | --- |
| MFI_min_^a^ | 1000 | 60 | 22 | 22 | 12 |
| MFI_mean_^b^ | 10328.18 | 2042.25 | 58.84 | 66.16 | 42.94 |

^a^Values are the minimum MFI value to each ACC10 in the whole subset. ^b^Values are the mean MFI value to each ACC10 in the whole subset. pro-RA: promiscuous rheumatoid arthritis patients’ sera; pro-OA: promiscuous osteoarthritis patients’ sera; pri-RA: private rheumatoid arthritis patients’ sera; pri-OA: private osteoarthritis patients’ sera; HC: healthy individuals’ sera.

Supplementary Table S3. Information of other antigen peptides.

| Name | Antigen | Post-translational modification |
| --- | --- | --- |
| fib cit 617-635 | fibrinogen | Citrullination |
| CEP-1 | α-enolase | Citrullination |
| CCP-1 | filaggrin | Citrullination |
| vim cit 58-77 | vimentin | Citrullination |

Supplementary Table S4. Triple-helical COL2 peptide sequences.

| Name | Epitope | mAb | Sequence |
| --- | --- | --- | --- |
| CII_T_E10_R | E10 | CIIE8, E10 (IL) | (GPO)_5_-GAEGPOGPQGLAGQRGIVGLOGQR-(GPO)_5_-K knot^bio^ |
| CII_T_C1_R-R | C1 | CIIC1, CB20 | (GPO)_5_-GDOGROGEOGLOGARGLTGROGDA-(GPO)_5_-K knot^bio^ |
| CII_T_E17 | E17 |  | (GPO)_5_-GTOGSOGPAGASGNOGTDGIOGAK-(GPO)_5_-K knot^bio^ |
| CII_T_J1_R | J1 | M2139 | (GPO)_5_-GAQGPOGLQGMOGERGAAGIAGPK-(GPO)_5_-K knot^bio^ |
| CII_T_F4_R-R(new) | F4 | CIIF4 | (GPO)_5_-GDKGEAGEOGERGLKGHRGFTGLQ-(GPO)_5_-K knot^bio^ |
| CII_T_E10_CIT | E10 | E10 | (GPO)_5_-GAEGPOGPQGLAGQ[Cit]GIVGLOGQR-(GPO)_5_-K knot^bio^ |
| CII_T_C1_CIT-R | C1 | ACC3, ACC4 | (GPO)_5_-GDOGROGEOGLOGA[Cit]GLTGROGDA-(GPO)_5_-K knot^bio^ |
| CII_T_C1_R-CIT | C1 | ACC1 | (GPO)_5_-GDOGROGEOGLOGARGLTG[Cit]OGDA-(GPO)_5_-K knot^bio^ |
| CII_T_C1_CIT-CIT | C1 | ACC1, ACC3 | (GPO)_5_-GDOGROGEOGLOGA[Cit]GLTG[Cit]OGDA-(GPO)_5_-K knot^bio^ |
| CII_T_U1_R-R | U1 | UL1 | (GPO)_5_-GDQGVOGEAGAOGLVGPRGERGFO-(GPO)_5_-K knot^bio^ |
| CII_T_D3_R | D3 | CIIC2 | (GPO)_5_-GPTGVTGPKGARGAQGPOGATGFO-(GPO)_5_-K knot^bio^ |
| CII_T_59 |  | PC12 | (GPO)_5_-GASGDRGPOGPVGPOGLTGPAGEO-(GPO)_5_-K knot^bio^ |
| CII_T_F4_CIT-CIT | F4 | CIIF4 | (GPO)_5_-GDKGEAGEOGE[Cit]GLKGH[Cit]GFTGLQ-(GPO)_5_-K knot^bio^ |
| CII_T_F4_R-CIT | F4 | CIIF4 | (GPO)_5_-GDKGEAGEOGERGLKGH[Cit]GFTGLQ-(GPO)_5_-K knot^bio^ |
| CII_T_F4_CIT-R | F4 | CIIF4 | (GPO)_5_-GDKGEAGEOGE[Cit]GLKGHRGFTGLQ-(GPO)_5_-K knot^bio^ |
| CII_T_U1_CIT-R | U1 | UL1 | (GPO)_5_-GDQGVOGEAGAOGLVGP[Cit]GERGFO-(GPO)_5_-K knot^bio^ |
| CII_T_U1_R-CIT | U1 | UL1 | (GPO)_5_-GDQGVOGEAGAOGLVGPRGE[Cit]GFO-(GPO)_5_-K knot^bio^ |
| CII_T_U1_CIT-CIT | U1 | UL1 | (GPO)_5_-GDQGVOGEAGAOGLVGP[Cit]GE[Cit]GFO-(GPO)_5_-K knot^bio^ |
| CII_T_J1_CIT | J1 | M2139 | (GPO)_5_-GAQGPOGLQGMOGE[Cit]GAAGIAGPK-(GPO)_5_-K knot^bio^ |
| CII_T_D3_CIT | D3 | CIIC2 | (GPO)_5_-GPTGVTGPKGA[Cit]GAQGPOGATGFO-(GPO)_5_-K knot^bio^ |
| CII_T_F4_R-R(old) | F4 | CIIF4 | (GPO)_5_-GDKGEAGEOGERGLKGHRGFTGLQ-(GPO)_5_-K knot^bio^ |

Supplementary Table S5. Peptides to which private sera have specific responses.

| Sera NO. | Peptide name | Citrulline site |
| --- | --- | --- |
| pri-RA1 | CII _C_12_CIT | 12cit |
| pri-RA2 | CII_C_(C1)_[CIT]-R | 19cit |
| pri-RA3 | CII _C_12_CIT | 12cit |
| pri-RA4 | CII_C_(E41)_[CIT]-R-(R) | 6cit |
| pri-RA5 | CII_C_(C1)_[CIT]-R | 19cit |
| pri-RA6 | CII_C_(E41)_[CIT]-R-(R) | 6cit |
| pri-RA7 | CII_C_U1_[CIT]-R | 25cit |
| pri-RA8 | CII _C_46_CIT | 46cit |
| pri-RA9 | CII _C_4_CIT | 4cit |
| pri-RA10 | CII_C_U1_[CIT]-R | 25cit |
| pri-OA1 | CII_C_(C1)_[CIT]-R | 19cit |
| pri-OA2 | CII _C_5_CIT | 5cit |
| pri-OA3 | CII_C_(E41)_[CIT]-R-(R) | 6cit |
| pri-OA4 | CII_C_U1_[CIT]-R | 25cit |
| pri-OA5 | CII_C_U1_[CIT]-R | 25cit |
| pri-OA6 | CII_C_(C1)_[CIT]-R | 19cit |
| pri-OA7 | CII _C_4_CIT | 4cit |
| pri-OA8 | CII_C_(C1)_[CIT]-R | 19cit |
| pri-OA9 | CII_C_(C1)_[CIT]-R | 19cit |
| pri-OA10 | CII_C_F4_R-[CIT] | 49cit |


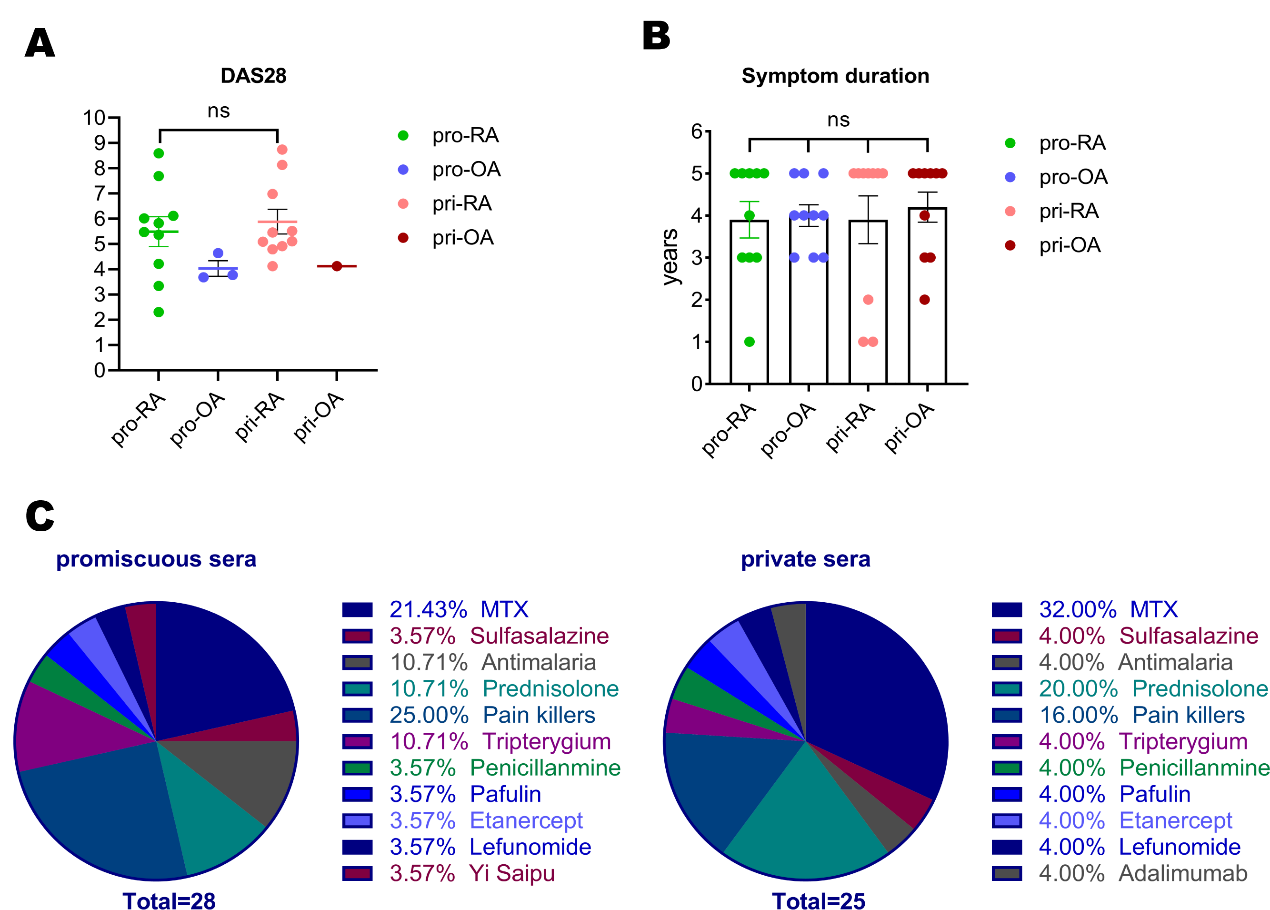


**Fig. S1 Some characteristics from patients with promiscuous or private ACPA**. **A** DAS28 assessment of patients. All RA patients’ DAS28 were collected. Only 4 DAS28 assessments were collected from OA patients (3 from pro-OA and 1from pri-OA). There is no significance between pro-RA and pri-RA groups. **B** The arthritic symptom duration from all 40 patients were recorded and no difference was found among groups. **C** The treatments to relieve arthritis are similar between promiscuous and private sera group. Only 2 OA patients (1 for each group) used treatments which were pain killers.


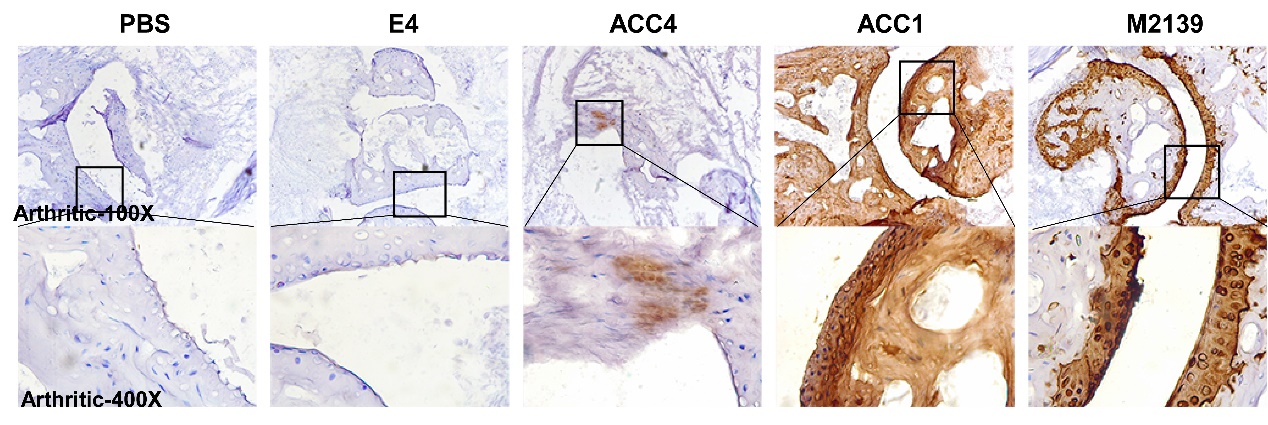


**Supplementary Figure S2.** Monoclonal ACPA and COL2 reactive antibody *in vitro* binding to newly developed arthritic joint tissue (arthritic score=13).
